# Supplementary figures and images for: Microbial Diversity and Community Variation in the Intestines of Layer Chickens
Source: Animals (Basel). 2021 Mar 16;11(3):840. doi: 10.3390/ani11030840 (PMC8002243; doi:10.3390/ani11030840)

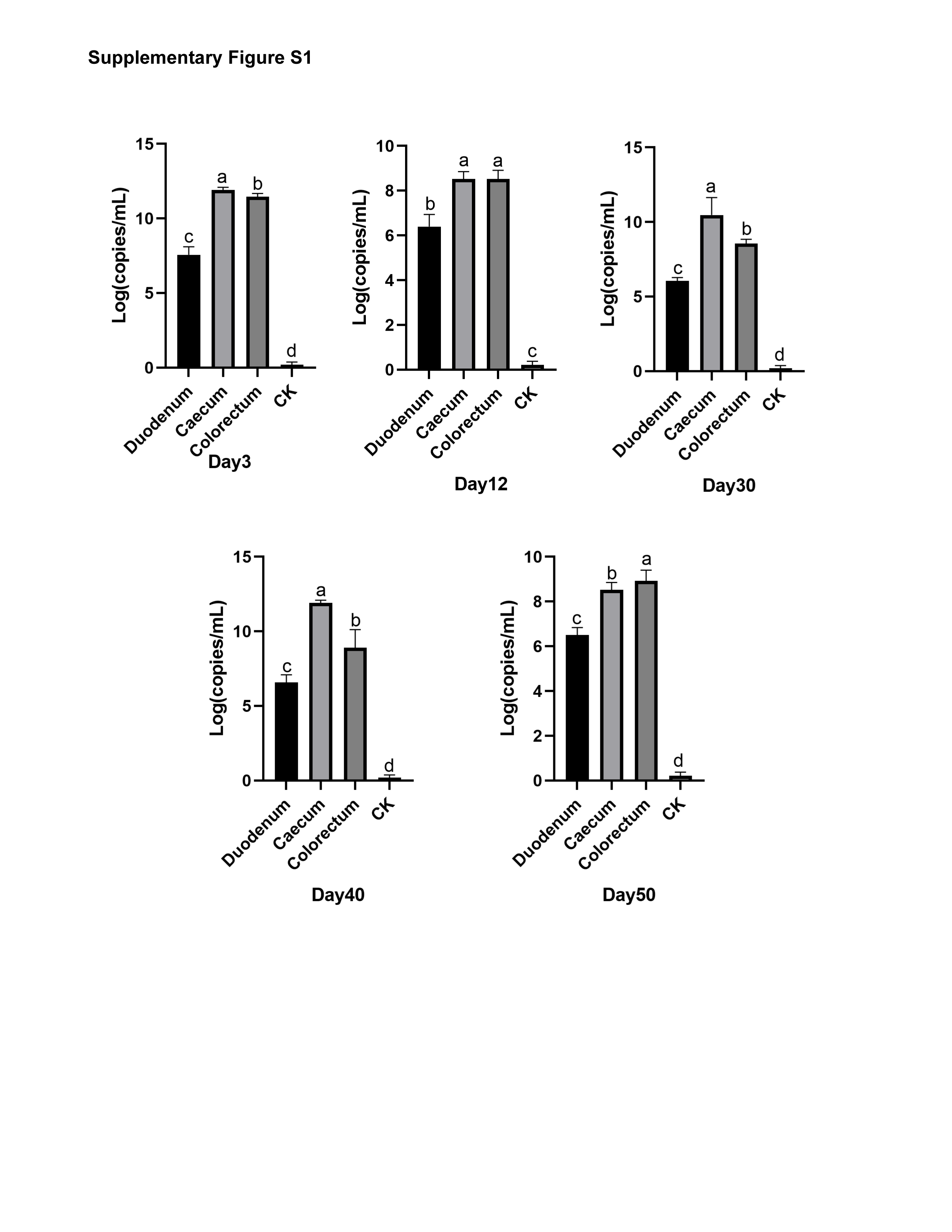

Supplement: Supplementary file 1 [file animals-11-00840-s001.zip › Supple mentary/Supplementary FigureS1.tif]

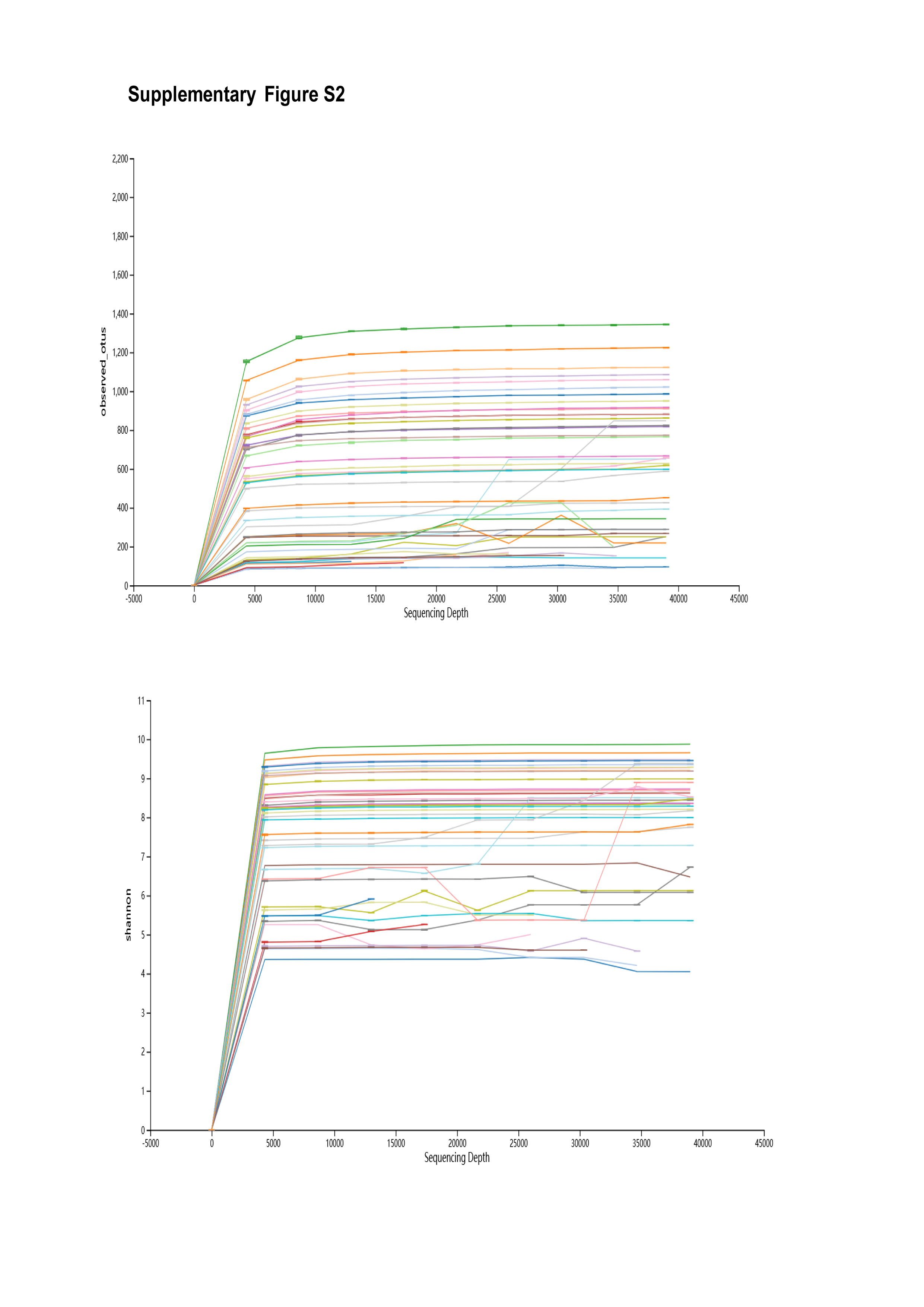

Supplement: Supplementary file 1 [file animals-11-00840-s001.zip › Supple mentary/Supplementary FigureS2.tif]

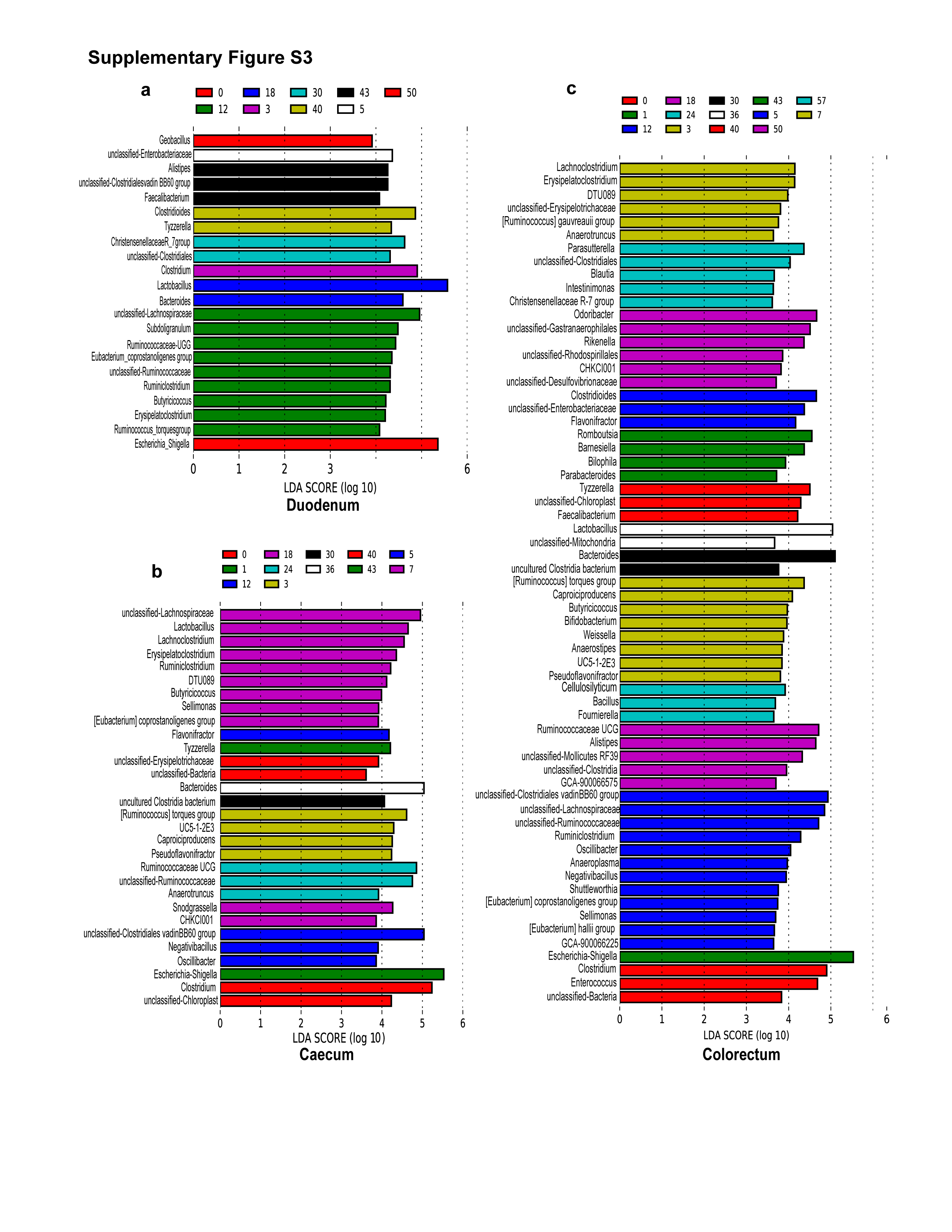

Supplement: Supplementary file 1 [file animals-11-00840-s001.zip › Supple mentary/Supplementary FigureS3.tif]

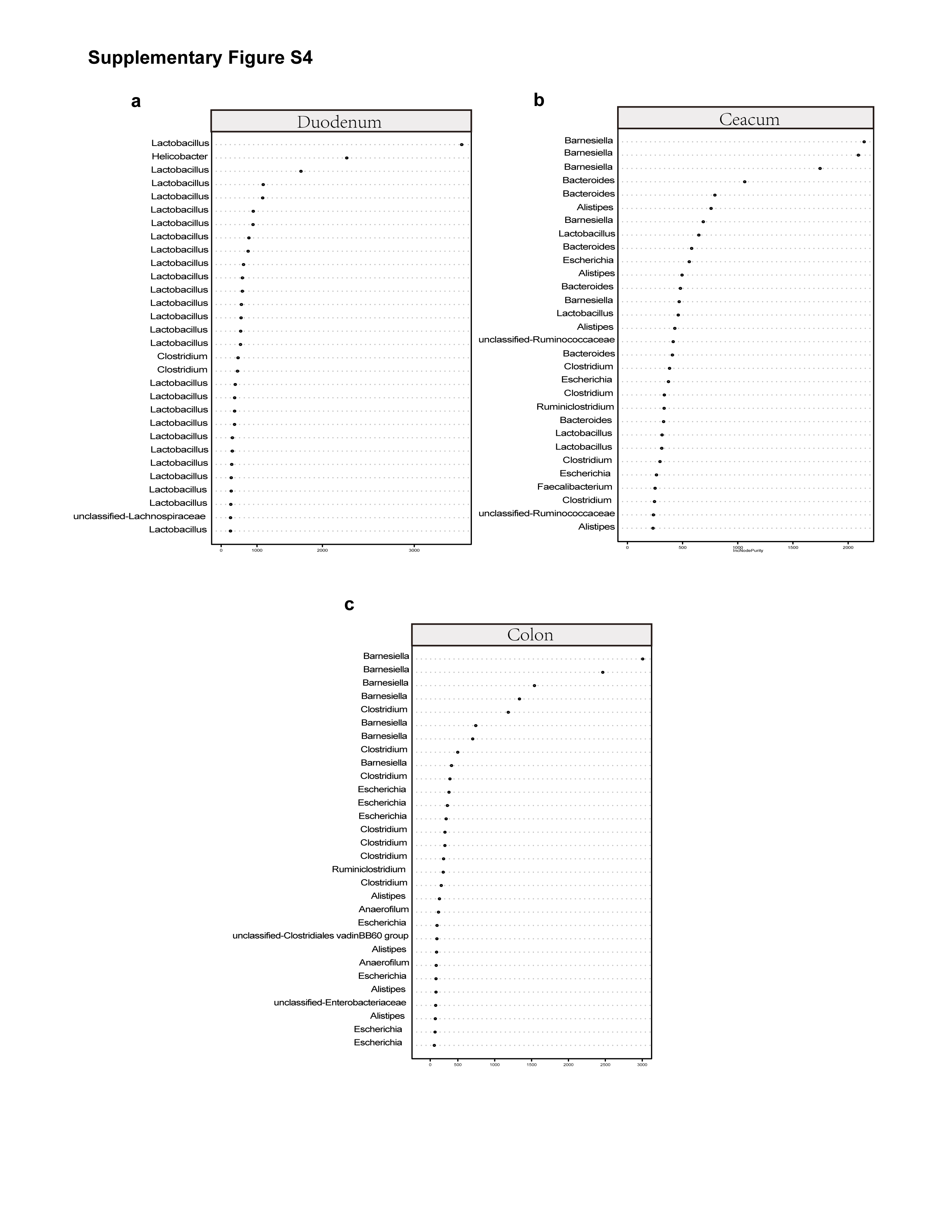

Supplement: Supplementary file 1 [file animals-11-00840-s001.zip › Supple mentary/Supplementary FigureS4.tif]

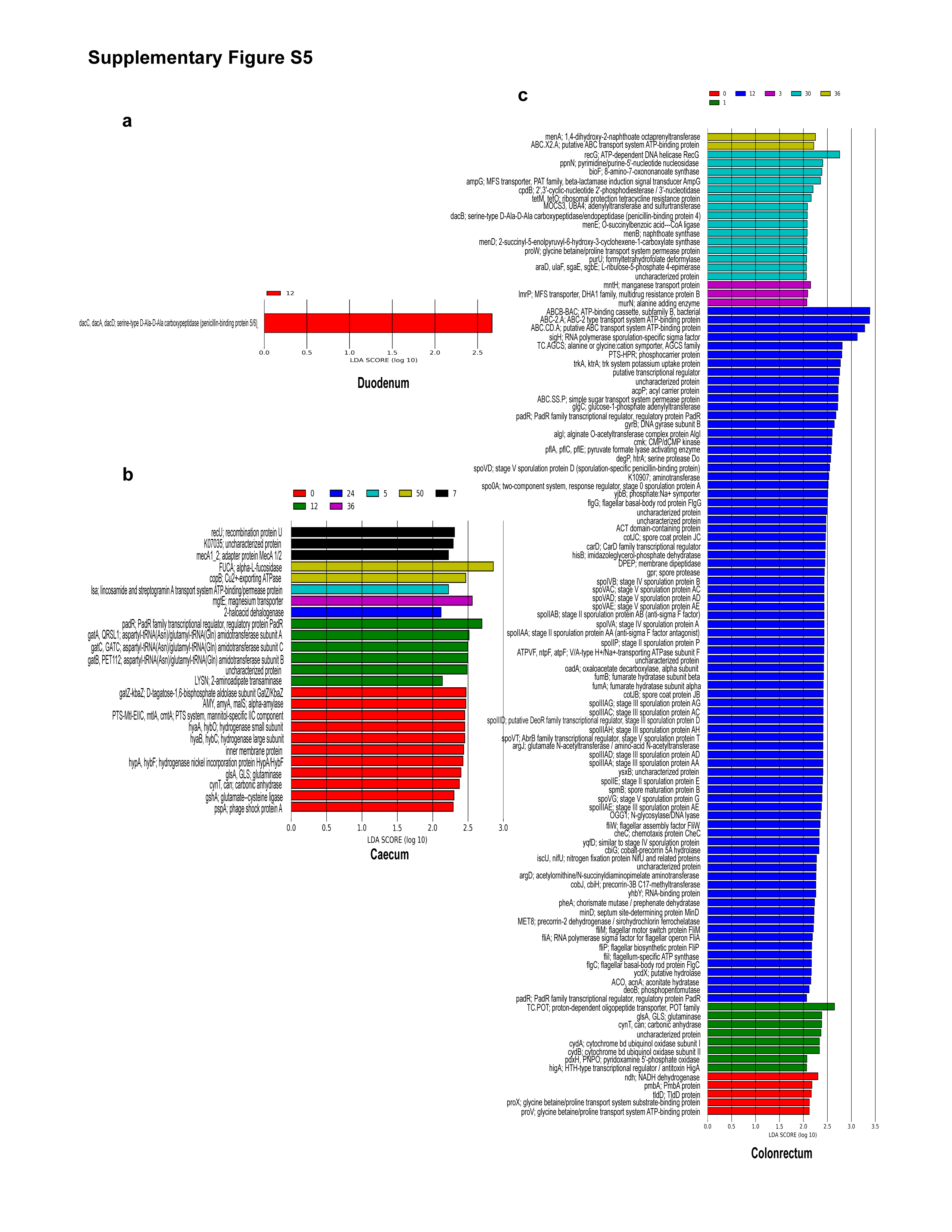

Supplement: Supplementary file 1 [file animals-11-00840-s001.zip › Supple mentary/Supplementary FigureS5.tif]

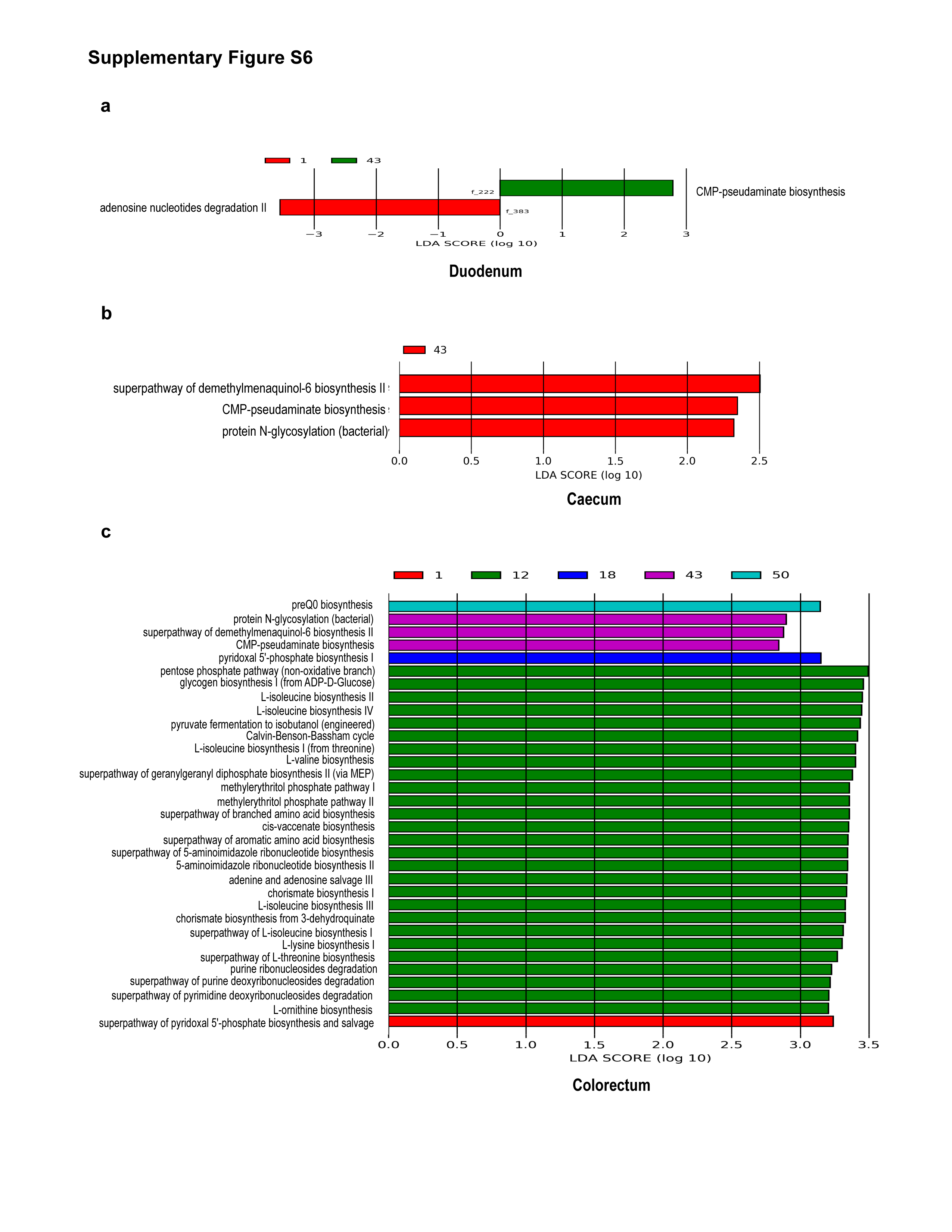

Supplement: Supplementary file 1 [file animals-11-00840-s001.zip › Supple mentary/Supplementary FigureS6.tif]
